# Analysis of international traveler mobility patterns in Tokyo to identify geographic foci of dengue fever risk

**DOI:** 10.1186/s12976-021-00149-8

**Published:** 2021-10-03

**Authors:** Baoyin Yuan, Hyojung Lee, Hiroshi Nishiura

**Affiliations:** 1 Graduate School of Medicine, Hokkaido University, Kita 15 Jo Nishi 7 Chome, Kita-ku, Sapporo-shi, Hokkaido 060-8638 Japan; 2 CREST, Japan Science and Technology Agency, Honcho 4-1-8, Kawaguchi, Saitama, 332-0012 Japan; 3 School of Mathematics, South China University of Technology, 381 Wushan Rd, Tianhe District, Guangzhou, China; 4 Department of Statistics, Kyungpook National University, Daegu, 41566 South Korea; 5 Kyoto University School of Public Health, Yoshidakonoecho, Sakyoku, Kyoto, 6068501 Japan

**Keywords:** Dengue, Imported case, Transmission, Tourism, Sightseeing, Coherence

## Abstract

Travelers play a role in triggering epidemics of imported dengue fever because they can carry the virus to other countries during the incubation period. If a traveler carrying dengue virus visits open green space and is bitten by mosquitoes, a local outbreak can ensue. In the present study, we aimed to understand the movement patterns of international travelers in Tokyo using mobile phone data, with the goal of identifying geographical foci of dengue transmission. We analyzed datasets based on mobile phone access to WiFi systems and measured the spatial distribution of international visitors in Tokyo on two specific dates (one weekday in July 2017 and another weekday in August 2017). Mobile phone users were classified by nationality into three groups according to risk of dengue transmission. Sixteen national parks were selected based on their involvement in a 2014 dengue outbreak and abundance of *Aedes* mosquitoes. We found that not all national parks were visited by international travelers and that visits to cemeteries were very infrequent. We also found that travelers from countries with high dengue prevalence were less likely to visit national parks compared with travelers from dengue-free countries. Travelers from countries with sporadic dengue cases and countries with regional transmission tended to visit common destinations. By contrast, the travel footprints of visitors from countries with continuous dengue transmission were focused on non-green spaces. Entomological surveillance in Tokyo has been restricted to national parks since the 2014 dengue outbreak. However, our results indicate that areas subject to surveillance should include both public and private green spaces near tourist sites.

## Introduction

Human mobility can be classified as migration (i.e., unidirectional movement) and circulation (i.e., departure and return after a first move away); these processes take place at different spatial and temporal scales [1]. Global travel of humans is recognized as the most critical pathway for the rapid spread of emerging infectious diseases [1–3]. International travel allows a disease, or sometimes even an epidemic, to be imported from endemic countries to a previously disease-free country [4, 5]. The population of the importing country is often fully susceptible and the vectorial capacities of arthropod vectors have increased in recent years because of climate change. Both factors increase the risk of imported dengue fever outbreaks in temperate countries [6]. In light of the importance of international mobility, [7] examined the transmissibility of infectious diseases (as measured by the basic reproduction number, *R*_0_, the average number of secondary cases generated by a single primary case) in relation to human movement and social settings of transmission (e.g., outdoor environments and households). Frequent travelers play a role in triggering imported epidemics because they can carry pathogens to other countries during their incubation period [8].

Despite the critical importance of human mobility in epidemiology, explicit accounting for human movement patterns is rarely achieved because of the absence of mobility data with high spatiotemporal resolution [9]. In recent years, mobile phones have been shown to be a useful data source, enabling cap;ture of human movement patterns based on call records and WiFi connectivity history and allowing statistical inference of human mobility patterns. Mobile phone data are considered to be more useful when an epidemic develops in populated areas with highly connected contact patterns [10]. Tracing 100,000 mobile phone users, [11] found a high degree of temporal and spatial regularity in their movement patterns. Individuals most often traveled over short distances and frequently commuted to a limited number of geographic locations; this information would be useful in preventing epidemics. The pros and cons of using mobile phone data in modeling infectious disease epidemics have been discussed [9]. Published applications include modeling of malaria [3], rubella [12], dengue [13], and Ebola transmission [14].

Although dengue fever is not endemic in Japan, the risk of autochthonous outbreaks of dengue is believed to have increased, as demonstrated by a 2014 transient epidemic in Yoyogi park, Tokyo [15, 16]. One of the underlying factors associated with imported dengue outbreaks in temperate countries is the increasing volume of Japanese travelers returning from dengue-endemic areas [17–19]. Another factor is the growing number of international travelers visiting Japan from other countries with dengue transmission. In Tokyo, national parks and cemeteries are the major habitats for mosquito species [20] including *Aedes albopictus*, a primary vector of dengue transmission in Japan [21]. If a traveler carrying dengue virus visits an open green space and is bitten by mosquitoes, a local outbreak can ensue. By objectively identifying mobility patterns of travelers from dengue endemic countries, entomological surveillance of *Aedes* species and associated prevention activities could be targeted to high-risk areas.

In the present study, we aimed to understand the movement patterns of international travelers in Tokyo using mobile phone data, with the goal of identifying geographical foci of dengue transmission. In addition to routine mosquito surveys of green space conducted in the summer, especially in national parks, we expect that mobility patterns of international travelers would be a useful source of information.

## Materials & Methods

### Data source: Mobile phones, dengue transmission and national parks

Many international travelers visiting Japan rent a mobile phone with WiFi capability. Nippon Telegraph and Telephone (NTT) DoCoMo is the most popular operator in Japan, covering 38.1% of domestic mobile phone users and approximately 20% of incoming international visitors. Once users are connected to WiFi networks, their locations are traced by the closest tower to the signal base station. Accordingly, NTT DoCoMo is able to tie location to mobile phone identity, which in turn can be matched with customer age and nationality. We obtained the deidentified NTT DoCoMo data for academic use and measured the spatial distribution of international visitors in Tokyo on two specific dates (one weekday, 3rd July and another weekday, 7th August, 2017). The data were provided as comma separated value files containing dates, areas, countries and cumulative numbers of people who visited each specific area during daylight hours (10 AM to 6 PM). The summary datasets by country are provided in the Online Supporting Material. Geographic area was coded using an eight-digit number representing two-dimensional geographic coordinates (longitude and latitude) [22] with a resolution of 1 square kilometer. In the subsequent analyses, we focused on central Tokyo at a 10 square kilometer resolution as described by four six-digit mesh codes (533935, 533936, 533945, and 533946).

The potential risk of international travelers carrying dengue virus was classified according to their nationality. We adhered to the classifications of countries by the Centers for Disease Control and Prevention [23] into three groups: (i) Group 1 countries with continuous risk of dengue transmission (*n*=9 among all countries with international mobile phone user records in Japan), (ii) Group 2 countries with occasional outbreaks in some regions (regional transmission) (*n*=4), and (iii) Group 3 countries with only sporadic cases (*n*=38).

The 2014 outbreak in central Tokyo occurred in Yoyogi park, a national park with substantial green space in a major metropolitan area. The park is located within 1 kilometer of Shibuya station, the third busiest train station in Tokyo, and later cases in the 2014 outbreak occurred in other national parks in Tokyo. Following the outbreak, it was demonstrated that *Aedes* mosquitoes were abundant in national parks. Since then, the Tokyo metropolitan government has continuously monitored mosquitoes each summer. In the present study, 16 public green spaces were selected in Tokyo: Yoyogi Park, Meiji Jingu Gaien, Shinjuku Chuo Park, Sotobori Park, Ueno Park, Toyama Park, Hibiya Park, Sarue Onshi Park, Hamarikyu Garden, Komazawa Olympic Park, Kasai Rinkai Park, Oi Wharf Central Bayside Park, Odaiba Kaihin-Koen, Aoyama Cemetery, Yanaka Cemetery, and Somei Cemetery. These sites were part of the 2014 outbreak or were subject to routine entomological surveys by the Tokyo Metropolitan Institute of Public Health [24]. The green spaces were overlaid with the geographic areas most often visited by international travelers. We compared the frequency of visits by international travelers to these parks and six other popular tourist sites: Senso-ji, Ginza, Skytree, Tokyo Tower, Tsukiji Market and the Imperial Palace [25].

### Statistical analysis

In the following analyses, we counted the frequencies of visits by travelers to understand travel patterns. More specifically, we analyzed traveler behaviors, in particular, visitation frequency was analyzed according to dengue transmission level in country of origin.

We calculated interrater reliability, defined as the extent of agreement between two or multiple raters [26, 27]. In principle, the term rater can represent any pair of research subjects. In the present study, two raters, denoted by rater A and rater B, were defined as travelers from foreign countries and national parks, respectively. Here binary responses of “Yes” and “No” represent the visiting status. For calculation of agreement measures, we first constructed 2-by-2 contingency tables describing how two rates A/B interacted with “Yes” and “No” responses (Table 1). The new notation of “Outcome A/outcome B” was introduced to indicate that outcome A is a response of rater A and outcome B is a response of rater B, respectively.

**Table 1:** Two-by-two table of *N* subjects evaluated by two raters and two response variables

		Rater A			
		Yes	No	Total	Marginal
Rater B	Yes	*a*	*b*	*a+b*	*P*_*1.*_*=*(*a+b*)/*N*
	No	*c*	*d*	*c+d*	*P*_*2.*_*=*(*c+d*)/*N*
	Total	*a+c*	*b+d*	*N*	
	Marginal	*P*_*.1*_*=*(*a+c*)/*N*	*P*_*.2*_*=*(*b+d*)/*N*		

Table modified from [28]

We employed four mathematical metrics to measure agreement between raters A and B (observed agreement, prevalence rate, Cohen’s kappa and Gwet’s AC1) [28]. Using Table 1, four agreement measures can be written explicitly. Observed agreement is calculated as

1 $${P}_a=\left(a+d\right)/N,$$

where *P*_*a*_ measures the number of times both raters classify response into either “Yes” or “No” (i.e, Yes/Yes or No/No), regardless of actual propensity or chance. Exclusively considering double-positive elements alone (i.e., Yes only), the trait prevalence [29] is calculated as

2 $${P}_b=a/N.$$

Cohen’s kappa is also calculated to adjust the chance agreement,

3 $$\kappa =\frac{P_a-{P}_{e(k)}}{1-{P}_{e(k)}},$$

where *P*_*e*(*k*)_ is the expected agreement by chance, representing the sum of the products of the marginal distributions (i.e., *P*_*e*(*k*)_ = *P*_1._ ∙ *P*_.1_ + *P*_2._ ∙ *P*_.2_), where marginal probabilities were defined as ${P}_{1.}=\frac{\left(a+b\right)}{N},{P}_{2.}=\frac{\left(c+d\right)}{N},{P}_{.1}=\frac{\left(a+c\right)}{N}\ \mathrm{and}\ {P}_{.2}=\frac{\left(b+d\right)}{N}$ .

Several thorough review studies described statistical biases and limitations of Cohen’s kappa [28, 30, 31]. Cohen’s kappa has been generally used because of a simple measure of agreement. However, there are two paradoxes associated with Cohen’s kappa described in [1, 2]: (1) A high agreement can lead to a low Cohen’s kappa; and (2) Unbalanced marginal distributions can give higher values of Cohen’s kappa than more balanced marginal distributions. We calculated an alternative agreement measure, Gwet’s AC1 [28, 30]. Gwet’s AC1 is a reasonable chance-corrected agreement coefficient to overcome the limitations of existing agreement measures. Thus, Gwet’s AC1 is calculated as follows.

4 $$AC1=\frac{P_a-{P}_{e\left(\gamma \right)}}{1-{P}_{e\left(\gamma \right)}},$$

where the chance agreement is given by ${P}_{e\left(\gamma \right)}=2\left(\frac{P_{1.}+{P}_{.1}}{2}\right)\left(1-\frac{P_{1.}+{P}_{.1}}{2}\right)$.

Interrater analysis was applied for three comparisons. We divided the central Tokyo represented by four six-digit mesh codes, i.e., 533935, 533936, 533945, 533946 to be smaller sub-regions with eight-digit mesh and we counted the total number of the sub-regions as the *N*. First, we assessed the agreement between national parks. For each sub-region coded using eight-digit number, if it overlaps with both the visiting mesh of travelers from some country recorded in the mobile phone data and the mesh where the park is located in, this mesh will be counted in the Yes/Yes category; if this sub-region overlaps with either the visiting mesh of travelers from some country or the mesh where the park is located in, it is Yes/No or No/Yes; if this sub-region overlaps with neither, then it is categorized as No/No. Second, we examined the coherence among the three groups of countries of traveler origin. Analogous 2-by-2 tables as above were created. Groups of countries were now the raters and the destinations of travelers from each group determined the category in which each geographic mesh would be counted. To be specific, we counted the number of sub-regions obtained in the abovementioned way: if one eight-digit mesh was visited by travelers from both groups of countries under consideration, it was counted as Yes/Yes; if this sub-mesh was visited by only one group of countries, it is Yes/No or No/Yes; otherwise, it was in No/No category. Third, we investigated the frequencies of international travelers who visited public green spaces and popular tourist sites. The 2-by-2 table for selected parks and popular tourist sites was filled by counting numbers of countries. We counted the number of countries from which travelers visited both the public green spaces and popular tourist sites to be Yes/Yes, visiting either the public green spaces or popular tourist sites to be Yes/No or No/Yes, visiting neither of the two spots to be No/No category.

### Ethical considerations

The datasets used in our study were fully anonymized and the analysis therefore did not require ethical approval.

### Data sharing

A summary dataset of the study population by country on single weekdays in July and August 2017 is provided in the Online Supporting Material.

## Results

**Fig. 1:**
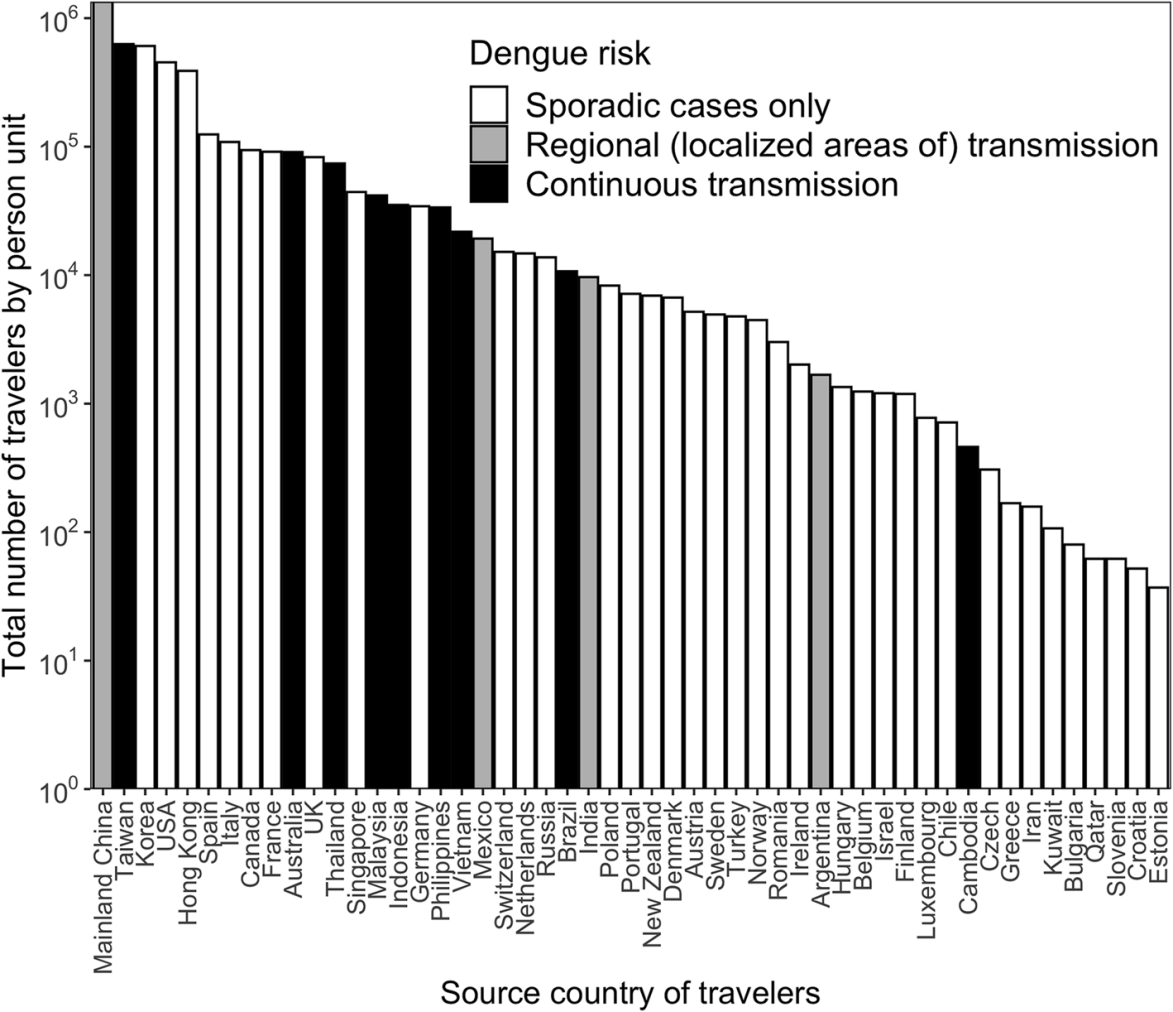
Country-specific numbers of international travelers in Tokyo, August 2017. The y-axis of the bar plot is on a log10 scale. According to the Centers for Disease Control and Prevention definitions of areas with dengue transmission risk, all 51 countries under consideration were classified into three groups: “sporadic cases only” in white, “regional (localized) transmission” in gray, and “continuous transmission” in black

**Fig. 2:**
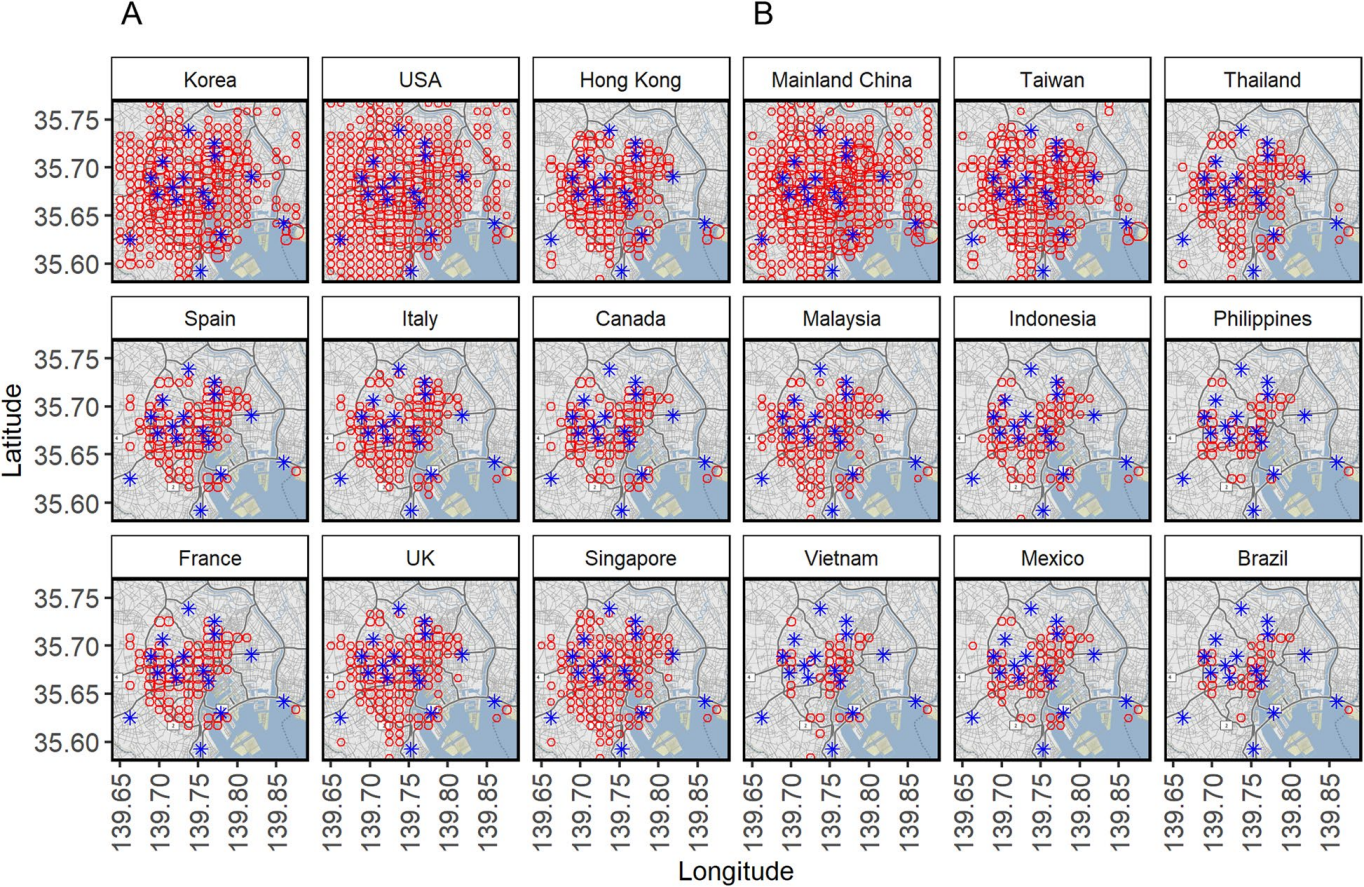
Spatial distribution of national parks and destinations of travelers by country in central Tokyo. In each panel, the horizontal axis shows longitude and the vertical axis shows latitude. The blue stars indicate the locations of national parks with potential risk of dengue transmission and the red circles represent the destinations of international travelers. While the nine countries on the left were dengue free (sporadic cases only), the remaining nine countries on the right were dengue endemic countries (regional transmission and continuous transmission)

In total, we analyzed the records of 9,688,405 travelers on one weekday in July 2017 and another weekday in August 2017. Fig. 1 shows the total numbers of international travelers according to their country of origin in August. The records of international travelers from a total of 51 countries were analyzed. The geographical locations of national parks and destinations of travelers from 18 countries (nine countries with and without dengue transmission, selected based on volume of travelers) are shown in Fig. 2. It should be noted that not all national parks are visited by international travelers. Visits to cemeteries were infrequent.

Table 2 summarizes agreement between the geographic locations of national parks and travel destinations. Gwet’s AC1 was higher among travelers from dengue-nonendemic countries compared with those from dengue-endemic countries. Because of the large number of travelers from Korea, the USA and mainland China (Fig. 1), AC1 took on negative values for these regions, reflecting difficulties in capturing the agreement between parks and visitation destinations. Overall, values of *P*_*b*_ were small between 0.01 and 0.04, representing the proportion of Yes/Yes category. In other words, the small observed agreement for Yes/Yes responses was seen between national parks and visit destinations of international travelers by country of origin. Given low value of Kappa and high value of AC1 for given small values of *P*_*b*_, the finding can be interpreted as an agreement that overrepresents high proportion of No/No category. This finding indicates that most travelers from countries with small values of *P*_*b*_ did not consistently choose national parks as their destination. On the basis of the four agreement measures, values for dengue-free countries (or those with sporadic cases; e.g., Korea, USA, Hong Kong, Spain, Italy, Canada, France, UK, and Singapore) and countries with regional and continuous dengue (e.g., Mainland China, Taiwan, Thailand, Malaysia, Indonesia, Philippines, Vietnam, Mexico, and Brazil) were comparable. The average AC1 value for the nine dengue-free countries was 4.1, lower than the average of 5.7 among countries with dengue. National parks showed higher agreement with visit destinations among travelers from countries with dengue. Comparing *P*_*a*_ and *P*_*b*_ between dengue-free and dengue-endemic countries, a smaller proportion of geographic meshes occupied by national parks and visit destinations were identified among the latter countries. This finding highlights the lower frequency of visits from travelers from countries with dengue to selected national parks.

**Table 2:** Agreement analysis between national parks and visit destinations of international travelers by country/region of origin

Traveler nationality	*P*_*a*_	*P*_*b*_	Kappa	AC1
Korea	0.41	0.04	0.04	-0.07
USA	0.27	0.04	0.02	-0.41
Hong Kong	0.68	0.03	0.09	0.54
Spain	0.78	0.03	0.14	0.71
Italy	0.77	0.03	0.13	0.69
Canada	0.83	0.03	0.17	0.78
France	0.81	0.03	0.17	0.76
UK	0.71	0.03	0.10	0.59
Singapore	0.69	0.03	0.10	0.55
Mainland China	0.35	0.03	0.02	-0.20
Taiwan	0.59	0.03	0.07	0.35
Thailand	0.74	0.03	0.12	0.64
Malaysia	0.75	0.03	0.12	0.65
Indonesia	0.83	0.03	0.17	0.79
Philippines	0.89	0.02	0.17	0.87
Vietnam	0.90	0.02	0.22	0.89
Mexico	0.85	0.02	0.16	0.82
Brazil	0.91	0.01	0.13	0.89

*Abbreviations*: *Korea* Republic of Korea, *USA* United States of America, *UK* United Kingdom of Great Britain and Northern Ireland. The agreement values of *P*_*a*_, *P*_*b*_, Kappa and AC1 were calculated for each country according to the distributions of traveler destinations and selected parks. Four two-level geographic meshes (10 *km* × 10 *km*) of central Tokyo were considered to include 400 three-level meshes (1 *km* × 1 *km*). Numbers of meshes occupied by national parks or visited by international travelers were counted

We also analyzed agreement between the geographic meshes of visit destinations among the three different groups of countries (Table 3). Countries with continuous transmission of dengue fever had the lowest coherence values for all four agreement measures. Cohen’s Kappa and AC1 were calculated as 0.39 and 0.39 comparing dengue-continuous and dengue-sporadic countries. In contrast, kappa and AC1 comparing dengue-sporadic and dengue-regional countries were calculated as 0.58 and 0.72, respectively, indicating that the travel patterns of individuals from these two groups of countries generally agreed well with each other. Travelers from dengue-sporadic and dengue-regional countries were more likely to visit the same geographic mesh codes than the other two pairs of count groups, as reflected by a larger *P*_*b*_ value (0.64 compared with 0.46).

**Table 3:** Agreement analysis between visit destinations of international travelers from countries/regions with or without dengue

Comparison pairs	*P*_*a*_	*P*_*b*_	Kappa	AC1
Countries with continuous transmission vs sporadic cases only	0.68	0.46	0.39	0.39
Countries with continuous transmission vs regional (localized) transmission	0.78	0.46	0.57	0.57
Countries with sporadic cases only vs regional (localized) transmission	0.83	0.64	0.58	0.72

Countries were divided into: (i) Group 1 countries with continuous risk of dengue transmission (*n*=9 among all countries with international mobile phone user records in Japan), (ii) Group 2 countries with occasional outbreaks in some regions (regional transmission) (*n*=4), and (iii) Group 3 countries with only sporadic cases (*n*=38). The agreement values of *P*_*a*_, *P*_*b*_, kappa and AC1 were calculated according to the distributions of geographic meshes visited by travelers. The numbers of meshes visited by travelers from each group of countries/regions were counted for further agreement analyses

**Fig. 3:**
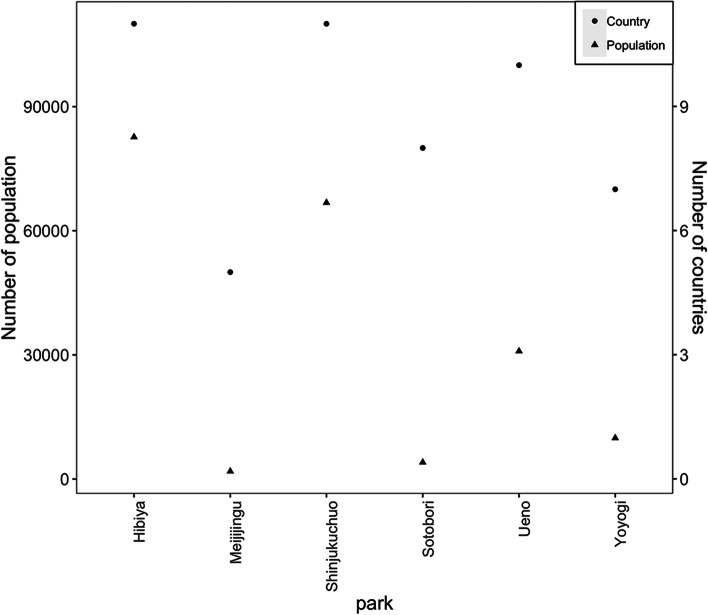
The numbers of travelers and countries by national park. Filled circles represent the number of the international travelers from dengue-positive countries by national park (measured in left vertical axis). The triangle shows the number of the countries from which international travelers arrived (measured in right vertical axis). Six parks were selected, including Hibiya Park, Meiji Jingu Gaien, Shinjuku Chuo Park, Sotobori Park, Ueno Park and Yoyogi Park

We subsequently examined agreement in each combination of parks (i.e., Yoyogi Park, Ueno Park, Sotobori Park and Meiji Jingu Gaien) and tourist sites (i.e. Senso-ji, Ginza, Skytree, Tokyo Tower, Tsukiji Market, and Imperial Palace) by counting the numbers of countries/regions for each combination (Table 4). Senso-ji and Ginza showed the lowest coherence values with national parks. Moreover, similar *P*_*a*_ and *P*_*b*_ values ranging from 0.38 to 0.77 indicated that almost half of travelers were unlikely to visit national parks if they visited Senso-ji or Ginza. Among the four national parks, the highest coherence with the six tourist sites was observed for Ueno park, which showed the highest values of Kappa and AC1 (0.51 and 0.83, respectively). Lower coherence measures for the other three parks suggested that travel patterns between these public green spaces and popular tourist sites were less correlated. We summarized the number of international travelers from dengue-positive countries and the number of countries of these travelers to six public parks in Fig. 3. Hibiya Park and Shinjuku Chuo Park were visited by the most travelers from dengue-positive countries, revealing the highest risk of the imported dengue virus, while the Meiji Jingu Gaien and Sotobori Park are the two parks attracting the least number of travelers from dengue-positive countries.

**Table 4:** Agreement analysis between national parks and tourist sites by international travelers

National park/tourist site combinations	*P*_*a*_	*P*_*b*_	Kappa	AC1
Yoyogi Park and Senso-ji	0.54	0.54	0	0.28
Yoyogi Park and Ginza	0.54	0.54	0	0.28
Yoyogi Park and Skytree	0.69	0.54	0.35	0.46
Yoyogi Park and Tokyo Tower	0.77	0.54	0.52	0.58
Yoyogi Park and Tsukiji Market	0.77	0.54	0.52	0.58
Yoyogi Park and Imperial Palace	0.69	0.54	0.35	0.46
Ueno Park and Senso-ji	0.77	0.77	0	0.71
Ueno Park and Ginza	0.77	0.77	0	0.71
Ueno Park and Skytree	0.92	0.77	0.75	0.89
Ueno Park and Tokyo Tower	0.85	0.69	0.57	0.76
Ueno Park and Tsukiji Market	1	0.77	1	1
Ueno Park and Imperial Palace	0.92	0.77	0.75	0.89
Sotobori Park and Senso-ji	0.62	0.62	0	0.44
Sotobori Park and Ginza	0.62	0.62	0	0.44
Sotobori Park and Skytree	0.54	0.38	0.20	0.12
Sotobori Park and Tokyo Tower	0.62	0.38	0.32	0.25
Sotobori Park and Tsukiji Market	0.62	0.38	0.32	0.25
Sotobori Park and Imperial Palace	0.54	0.38	0.20	0.12
Meiji Jingu Gaien and Senso-ji	0.38	0.38	0	-0.07
Meiji Jingu Gaien and Ginza	0.38	0.38	0	-0.07
Meiji Jingu Gaien and Skytree	0.54	0.38	0.20	0.12
Meiji Jingu Gaien and Tokyo Tower	0.62	0.38	0.32	0.25
Meiji Jingu Gaien and Tsukiji Market	0.62	0.38	0.32	0.25
Meiji Jingu Gaien and Imperial palace	0.54	0.38	0.20	0.12

The agreement values of *P*_*a*_, *P*_*b*_, kappa and AC1 were calculated according to the distributions of the numbers of origin countries of travelers visiting national parks or popular tourist sites. Here only the countries with continuous or regional transmission of dengue were considered in this table

**Fig. 4:**
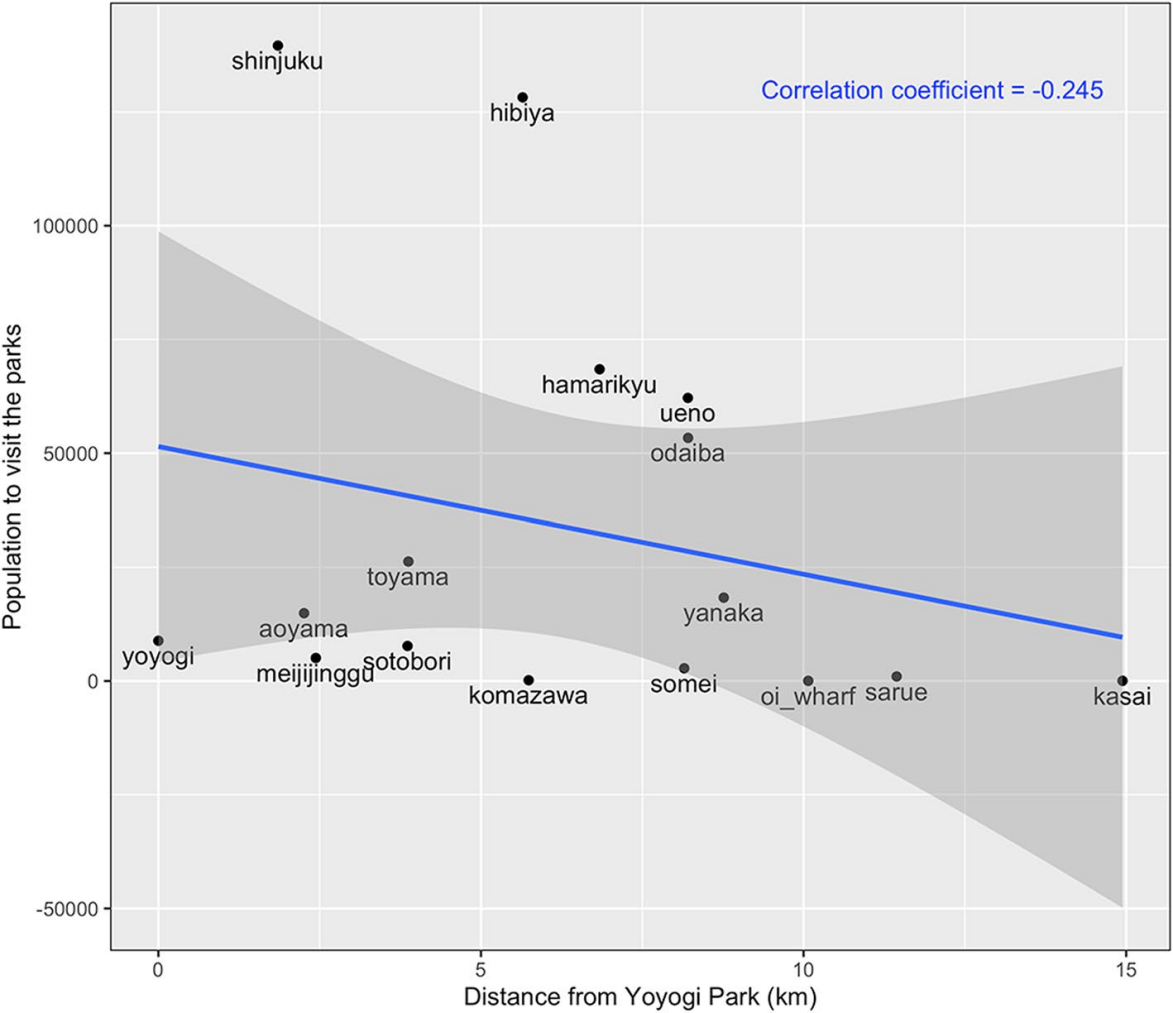
The correlation between the number to visitors to sixteen selected parks and the relative distance from Yoyogi Park to other fifteen parks. The X-axis is the relative distance in kilometers from Yoyogi Park to other fifteen parks including Meiji Jingu Gaien, Shinjuku Chuo Park, Sotobori Park, Ueno Park, Toyama Park, Hibiya Park, Sarue Onshi Park, Hamarikyu Garden, Komazawa Olympic Park, Kasai Rinkai Park, Oi Wharf Central Bayside Park, Odaiba Kaihin-Koen, Aoyama Cemetery, Yanaka Cemetery, and Somei Cemetery. The Y-axis shows the total number of travelers from dengue-positive countries who visited each park. The blue line is the linear regression line. The correlation coefficient was calculated by the Pearson’s moment correlation

In Fig. 4, we calculated the correlation coefficient between the population size of visitors to the 16 selected parks and the relative distance from Yoyogi Park to other 15 parks. The correlation coefficient was estimated at -0.175, revealing the low correlation between tourists in choosing the closer places, i.e., the geographic distance was not an important factor to affect the travelers’ choice of parks.

## Discussion

In the present study, we analyzed the geographic mobility patterns of international travelers visiting Tokyo, Japan using mobile phone data. Using four coherence measures, we compared (1) the locations of national parks and visit destinations, (2) visit destinations of travelers among three groups of countries with different transmission levels of dengue, and (3) visit experiences of travelers to four national parks and six popular tourist sites. We found that travelers from dengue-positive countries were less likely to visit national parks compared with travelers from dengue-free countries. Travelers from countries with sporadic cases and countries with regional transmission visited common destinations, while the travel footprints of individuals from countries with continuous transmission were focused on non-green spaces. Among travelers from countries with dengue, limited coherent travel patterns were observed when comparing national parks and popular tourist sites.

To our knowledge, our study is the first to analyze the travel patterns of visitors from various countries/regions in Tokyo. Considering that imported dengue cases and outbreaks are always triggered by human movement from countries with dengue to dengue-free destination countries, tracing the movements of travelers and identifying geographic risk hotspots for outbreaks is vital. For current mosquito surveillance efforts, areas of investigation are focused on public green space, mostly national parks [24]. However, our data suggest that not all national parks, especially cemeteries, are at high risk of dengue transmission from international visitors. In addition to mosquito abundance, the risk of a potential outbreak is expected to depend on the number of visitors from countries with dengue. Low coherence was observed between park locations and visit destinations among travelers from dengue-endemic countries. Given this finding, candidate areas for entomological surveillance should be explored in a broader scope, balancing frequently visited locations with areas having a substantial abundance of *Aedes* mosquitoes. Heterogeneous coherence between selected parks and tourist sites implies that both public and private green space nearby tourist sites could be potential areas of interest.

For this type of exercise to more explicitly map the geographic risk of dengue, modeling of vectorial capacity should be included. Instead of using the locations of national parks as the benchmark, it would then be possible to describe the reproductive number as a function of temperature and mosquito abundance across space and time; these data could then be overlaid on to the population sizes of international travelers. However, mosquito abundance in central Tokyo is restricted to very localized areas and conditions (e.g., green spaces with standing water). Thus, a sensible next step would be to model land use at higher resolution near tourist areas to identify hotspots of *Aedes* mosquitoes at a local level.

Our study had at least four technical limitations. First, in our agreement analysis of visit destinations, we counted common destinations through space shared by travelers of different nationalities, ignoring the numbers of travelers of each nationality. The number of travelers visiting an identical geographic mesh code may vary by nationality. This assumption caused countries with small numbers of travelers to have the same weight as countries with greater numbers of travelers, possibly increasing coherence levels. Second, to create the contingency table of national parks and popular tourist sites (Table 4), we counted the numbers of involved countries instead of the volume of travelers by country. This simplification ignored the effect of country-specific populations, which could again have led to overestimated coherence values. Third, we used mobile phone data only from August 2017, so our results might not be generalizable across time. In particular, post-coronavirus disease 2019 epidemic travel patterns may involve very different tourist behaviors, and thus additional surveys must be planned. Fourth, we restricted the study area to central Tokyo. Other peripheral regions also attract tourists, and so designing an improved risk assessment method for such areas would be a significant improvement.

The present study identified mobility patterns of international travelers from various countries in Tokyo using mobile phone data. We found that travelers from dengue endemic countries were unlikely to visit national parks and to travel to both parks and tourist sites. Entomological surveys should not be restricted to national parks. Public or private green space near tourist sites could be potential areas of interest.
